# *Puerarin* Attenuates Cerebral Damage by Improving Cerebral Microcirculation in Spontaneously Hypertensive Rats

**DOI:** 10.1155/2014/408501

**Published:** 2014-02-13

**Authors:** Xu-Dong Wu, Chen Wang, Zhen-Ying Zhang, Yan Fu, Feng-Ying Liu, Xiu-Hua Liu

**Affiliations:** ^1^Out-Patient Department, Chinese PLA General Hospital, Beijing 100853, China; ^2^Department of Pathophysiology, Chinese PLA General Hospital, Beijing 100853, China; ^3^State Key Laboratory of Kidney Disease, Chinese PLA General Hospital, 2011DAV00088, Beijing 100853, China

## Abstract

*Puerariae Lobatae Radix* (Gegen in Chinese) is the dried root of *Pueraria lobata*, a semiwoody, perennial, and leguminous vine native to China. *Puerarin* is one of the effective components of isoflavones isolated from the root of *Pueraria lobata*. Previous studies showed that extracts derived from the root of *Pueraria lobata* possessed antihypertensive effect. Our study is to investigate whether *puerarin* contributes to prevention of stroke by improving cerebral microcirculation in rats. *Materials and Methods*. Video microscopy and laser Doppler perfusion imaging on the pia mater were used to measure the diameter of microvessel and blood perfusion in 12-week old spontaneously hypertensive rats (SHRs) and age-matched normotensive WKY rats. Histological alterations were observed by hematoxylin and eosin staining, and microvessel density in cerebral tissue was measured by immunohistochemical analysis with anti-Factor VIII antibody. Cell proliferation was detected by [^3^H]-TdR incorporation, and activities of p42/44 mitogen activated protein kinases (p42/44 MAPKs) were detected by western blot analysis in cultured cerebral microvascular endothelial cells (MECs). *Results*. Intravenous injection of *puerarin* relaxed arterioles and increased the blood flow perfusion in the pia mater in SHRs. *Puerarin* treatment for 14 days reduced the blood pressure to a normal level in SHRs (*P* < 0.05) and increased the arteriole diameter in the pia mater significantly as compared with vehicle treatment. Arteriole remodeling, edema, and ischemia in cerebral tissue were attenuated in *puerarin*-treated SHRs. Microvessel density in cerebral tissue was greater with *puerarin* than with vehicle treatment. *Puerarin*-treated MECs showed greater proliferation and p42/44 MAPKs activities than vehicle treatment. *Conclusions*. *Puerarin* possesses effects of antihypertension and stroke prevention by improved microcirculation in SHRs, which results from the increase in cerebral blood perfusion both by arteriole relaxation and p42/44 MAPKs-mediated angiogenesis.

## 1. Introduction

Hypertension is the key risk factor for stroke, the second leading cause of death in China, and is characterized by increased peripheral vascular resistance and microvascular abnormalities [1, 2]. When antihypertensive drugs reduce systemic blood pressure, the microvessel dysfunction contributes to decreased perfusion pressure in the brain [3]. The decreased perfusion pressure impairs blood perfusion in cerebral tissue and might lead to stroke. Hence, identifying novel therapies for more effective and safer antihypertensive drugs with microcirculation improvement in patients with imminent stroke is urgently needed.

*Pueraria Lobata Radix* (Gegen in Chinese) is the dried root of *Pueraria lobata* (Wild.) *Ohwi*, a semiwoody, perennial, and leguminous vine native to China, which has been used as an herbal medicine for the treatment of fever, diarrhea, headache, and cardiovascular diseases for more than 2000 years [4]. *Puerarin* is the first identified effective component from the root of *Pueraria Lobata Radix* [5] and has been used as the marker compound in herb quality evaluation in the Chinese Pharmacopoeia [6]. The other major ingredients of *Pueraria Lobata Radix* include flavonoid-including daidzin, daidzein, daidzein-4-7-glucoside, puerarin-7-xyloside, 4-6-O-acetyldaidzin, and alkaloid [7]. Total flavonoids of *pueraria* reduced blood pressure in a dose-dependent manner in spontaneously hypertensive rats (SHRs) [8], which indicates that* pueraria* reduces blood pressure and morbidity of stroke.

*Puerarin* injection has been widely employed in China for the treatment of acute ischemic stroke [4]. However, few reports are available on the mechanisms underlying stroke prevention of *Puerarin*. We hypothesized that *puerarin* contributes to the prevention of stroke by improving cerebral microcirculation and protecting brain against ischemia damage. We investigated the effects of *puerarin* both on pia mater microcirculation in SHRs and in cultured cerebral microvascular endothelial cells (MECs) by investigating the proliferation effects and its signaling pathway. We aimed to explain the mechanism by which *puerarin* contributes to prevention of stroke through improving cerebral microcirculation.

## 2. Material and Methods

### 2.1. Animals and Chemicals

#### 2.1.1. Animals

Twelve-week-old SHRs and age-matched normotensive Wistar-Kyoto (WKY) rats were housed 4 per cage, at 23°C, with luminosity cycles of 12 h light/12 h dark, fed regular rat chow, and allowed free access to water. The rats were provided by the Beijing Institute of Experimental Animals. All procedures were performed in accordance with the Guide for the Care and Use of Laboratory Animals published by the US National Institutes of Health (NIH Publication number 85-23, revised 1996) and approved by the local animal care and use committee.

#### 2.1.2. Chemicals

*Puerarin* was purchased from the Beijing Union Pharmaceutical Factory (Beijing, China). M199 medium was from Gibco Co. (Carlsbad, CA, USA). Endothelial cell growth supplement (ECGS), collagenase I, tetramethyl ethylene diamine (TEMED), phenylmethyl sulfonylfluoride (PMSF), sodium dodecyl sulphate (SDS), *β*-glycerophosphate, sodium orthovanadate, leupeptin, DTT, and anti-Factor VIII antibody were from Sigma Chemical Co. (St. Louis, MO, USA). Antibodies for total and phospho-p42/p44-MAPKs were from Santa Cruz Biotechnology. PD98059 was from Calbiochem. Prestained protein marker was from Bio-Rad Co. Antibodies specific for nonphosphorylated and phospho-specific p42/p44-MAPKs and avidin-biotin-peroxides complex (ABC) kit for immunohistochemistry were from Santa Cruz Biotechnology (Santa Cruz, CA, USA). Low-molecular-weight calibration kit for SDS electrophoresis was from Amersham Biosciences Co. (Buckinghamshire, UK); cocktail tablets were from Roche Co. (Basel, Switzerland).

### 2.2. Microscopy Monitoring of Pia Mater Microcirculation

After a 5-day acclimatization, a 4 mm diameter cranial window was drilled, and cerebral dura mater was separated in rats under anesthesia with pentobarbital sodium (30 mg/kg) as described previously [9]; the microcirculation of pia mater was monitored by use of a biomicroscope (BH-2, Olympus, Japan) equipped with a video camera (BY-110, JVC, Japan) and a computer. Blood flow perfusion of the cerebral pia mater was detected by use of Laser Doppler Perfusion Imager (LISCAPIMII, Sweden). After baseline monitoring, rats received *puerarin* (100 mg/kg) in 20% propanediol by tail-vein injection, and microcirculation and blood perfusion were monitored at 3, 5, 10, 15, 20, 30, and 40 min after *puerarin* injection.

### 2.3. Hemodynamic Measurements

Twelve-week-old male SHRs and age-matched normotensive WKY rats were housed and fed for 1 week and randomly divided into 7 groups (*n* = 10) for treatment: *puerarin*-treated SHRs and WKY rats, intraperitoneal injection of 100 mg/kg *puerarin* in 0.3 mL of vehicle (20% propanediol) every day for 14 days; vehicle-treated SHRs and WKY, the same volume (20%) of propanediol injected every day for 14 days; age-matched control SHRs and WKY rats, rats housed and fed routinely for 14 days; and nimodipine, SHRs receiving 30 mg/kg nimodipine by gastrogavage every day for 14 days. All rats were anesthetized with 20% urethane (10 mL/kg) by intraperitoneal injection 1 hr after the last treatment (or the same time in controls). Microcirculation and blood flow perfusion of cerebral pia mater were monitored as described above. Then mean arterial pressure (MAP) and heart rate were measured for consecutive 3 min in anesthetized rats with the right carotid artery cannulated and connected to a pressure transducer in line to a Grass polygraph (SMUP-PCI).

### 2.4. Immunohistochemical and Histological Staining of Cerebral Tissue

At the end of the experiment and within 3 min of sacrifice, brains were removed from rats, 2 coronal slices were made at 5 and 7 mm from the frontal pole, and brain slices were immersed in 10% phosphate-buffered formalin, routinely processed, and embedded in paraffin. Serial sections (6 *μ*m) were stained by hematoxylin and eosin for morphological assessment or dipped in cold acetone for 10 min for immunohistochemical staining [10]. To reduce nonspecific reactivity, sections were preincubated with 0.3% hydrogen peroxide and normal goat serum. Sections were incubated at 4°C overnight with rabbit antihuman Factor VIII-related antigen (1 : 200 dilution), a marker of endothelial cells, and then for 1 hr at 37°C with goat anti-rabbit immunoglobulin (1 : 500 dilution). Antibody-biotin conjugate was detected with use of a DAB (3,3N-Diaminobenzidine Tertrahydrochloride) kit according to the manufacturer's instructions. Microvessels were identified as being between 10 and 25 *μ*m. We randomly chose 10 fields at 50x magnification for microvessel counting; the number of microvessels is presented as number per mm^2^.

### 2.5. Culture of MECs

The MECs from SHRs and WKY rat pallium were cultured as described [11] with modification. Briefly, brain cortex was obtained from urethane-anesthetized SHRs or WKY rats. The pallium was fully minced, and cerebral microvessels were collected by filtrating the homogenate in medium 199 (M199) through 300 and 60 *μ*m nylon mesh screens consecutively. The filtrate was collected and suspended in M199 medium containing 2 mmol/L L-glutamine, 100 U/mL penicillin, 100 *μ*g/mL streptomycin, 25% fetal bovine serum (FBS), 40 U/mL heparin, and 100 *μ*g/mL ECGS. The suspension was cultured in a humidified atmosphere with 5% CO_2_ at 37°C. MECs were identified by their morphology under phase-contrast microscopy and by Factor VIII-related antigen. All individual batches of MECs were prepared from a single rat pallium. All experiments were performed on confluent quiescent MECs at the third or fourth passage and were exposed to serum-depleted medium (0.1% FBS) for 12 hr before the experiment.

### 2.6. Assessment of Cell Proliferation by [^3^H]-TdR Incorporation

Cells were plated in 24-well plates at 10^4^ cells/well in M199 supplemented with 10% FBS and 100 *μ*g/mL ECGS. After 24 hr incubation, MECs were rinsed with phosphate-buffered saline and incubated in 0.1% FBS and ECGS-deprived M199 for 12 h. Then, MECs from SHRs and WKY rats were randomly divided into experimental groups for treatment: *puerarin*, incubated with *puerarin* (25, 50, and 100 ng/L) for 24 h; control, incubated with vehicle (20% propanediol) for 24 hr; FBS, MECs from SHRs incubated with 10% FBS for 24 hr; PD98059+*puerarin*, *pretreated* with PD98059 (MEK-1/2 inhibitor, 50 *μ*mol/L) for 10 min before *puerarin* (100 ng/L) treatment; and BDM+*puerarin*, pretreated with an activator of protein phosphatase, 2,3-butanedione monoxide (BDM, 20 mmol/L) 10 min before *puerarin* (100 ng). Then cultures were incubated with 1 *μ*Ci [^3^H]-TdR (Amersham, USA) for 12 hr [12]. At the end of incubation, cells were washed twice with 4°C PBS and detached from wells by trypsinization. Detached MECs were harvested onto glass fiber filter paper by use of a mini-MASH II microharvesting device (Whittaker MA Bio-products, Walkesville, MD), and [^3^H]-TdR incorporated into MECs was determined by use of a liquid scintillation counter (Beckman LS 6500).

### 2.7. Western Blot Analysis of ERKs

MECs were rinsed and homogenized in ice-cold extraction buffer containing (in mmol/L) Hepes 20 (pH 7.7), MgC1_2_ 2.5, EDTA 0.1, *β*-glycerophosphate 20, DTT 0.5, sodium orthovanadate 0.1, NaCl 75, Leupeptin 4 (*μ*g/mL), PMSF 20 (*μ*g/mL), and triton X-100 0.05% (v/v). The homogenate was incubated and centrifuged at 4°C. Protein concentration in the detergent soluble supernatant was determined by use of a bicinchoninic acid protein assay kit according to the manufacturer's protocol. The supernatant was mixed with Laemmli buffer and heated for 5 min at 95°C. Soluble extracts (50 *μ*g) were loaded per lane and separated by SDS-PAGE and then electrophoretically transferred to a polyvinylidene difluoride filter membrane (0.45 *μ*m) and blocked with 10% nonfat dry milk. The blots were then incubated with the antibodies against total and phospho-p42/p44-MAPKs at 1 : 1000 dilution in TBS-T and then horseradish peroxidase-conjugated anti-rabbit IgGin TBS-T [13]. Antibody-antigen complexes were visualized by enhanced chemiluminescence. Membranes were exposed to X-ray film for 30 s to 2 min. The staining was quantified by scanning the films, and the band density was determined with use of Image-Pro software.

### 2.8. Statistical Analysis

Data is expressed as mean ± SD from at least 3 (immunohistochemical, immunoblotting, and histological staining) or 7 (measurement of hemodynamics and microcirculation) independent experiments. Differences in means between groups were tested by one-way ANOVA. *P* < 0.05 was considered statistically significant. All statistical analyses involved use of SPSS v12.0 (SPSS Inc., Chicago, IL).

## 3. Results

### 3.1. *Puerarin* Lowered Blood Pressure in SHRs

Hemodynamic data is shown in Table 1. The groups did not differ in heart rate (*P* > 0.05). The MAP was higher for SHRs than for WKY rats treated with vehicle (*P* < 0.05), with no significant differences in MAP for these rats than their controls (*P* > 0.05), respectively, which indicates that vehicle injection did not affect blood pressure of rats. WKY rats treated with or without *puerarin* did not differ in MAP (*P* > 0.05), which suggests that *puerarin* did not affect blood pressure in normotensive WKY rats. However, as compared with vehicle-treated SHRs, *puerarin*-treated SHRs showed significantly lower MAP, by 19.2% (*P* < 0.05), which was similar to that for WKY control rats (*P* > 0.05), which indicates that *puerarin* had an antihypertensive effect in SHRs.

### 3.2. *Puerarin* Attenuated Cerebral Injury in SHRs

**Figure 1:**
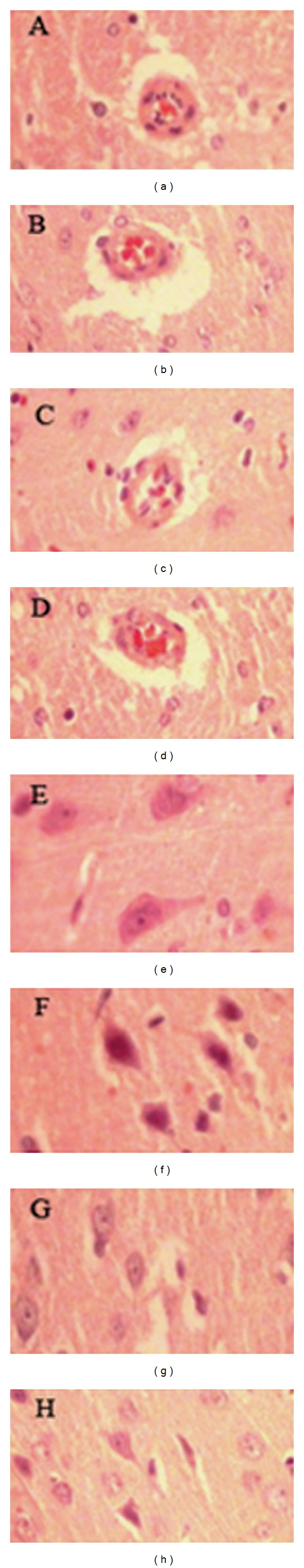
Effect of *puerarin* on brain microvascular remodeling in rats. (a)–(d) Brain microvascular remodeling. (a) Wistar-Kyoto (WKY) rats, (b) spontaneously hypertensive rats (SHRs), and (c) SHRs receiving *puerarin* (100 mg/kg *puerarin*) for 14 days by intraperitoneal injection. (d) SHRs receiving nimodipine (30 mg/kg) by gastrogavage for 14 days (serial sections (6 *μ*m) were stained by hematoxylin and eosin ((h)–(e)) as described in text, ×100).

H-E staining revealed normal structure in neurons and the arteriole wall in WKY rats but remodeling and hyaline degeneration in the arteriole wall (Figures 1(a)–1(d)) and regional ischemic neurodegeneration, edema, and neuron karyopyknosis (Figures 1(e)–1(h)) in SHRs, which regularly expanded to the basal ganglia and the neocortex. *Puerarin* treatment for 14 days attenuated these morphological alterations as compared with vehicle treatment.

### 3.3. *Puerarin* Treatment Increased Microvessel Density in Cerebral Tissue

**Figure 2:**
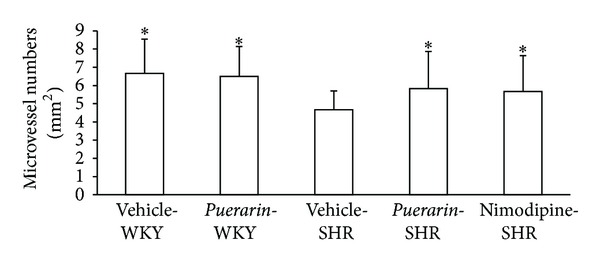
Effect of *puerarin* on brain microvessel density in rats. *P < 0.05 compared with SHRs receiving vehicle (20% propanediol).

Immunohistochemistry revealed microvessel density in cerebral tissue significantly increased in SHRs with *puerarin* treatment for 14 days as compared with propanediol-treated and control SHRs (Figure 2).

### 3.4. *Puerarin* Treatment Improved Pia Mater Microcirculation

**Figure 3:**
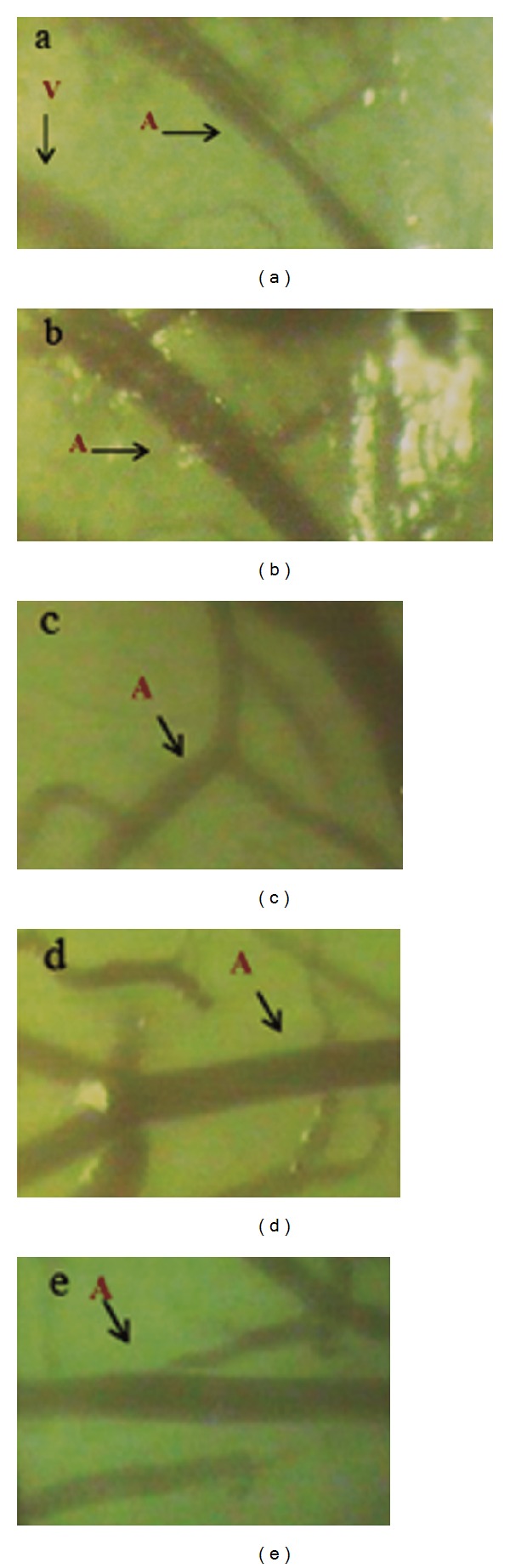
Effect of* puerarin* on microvessel diameter in pia mater of SHRs. (a) Before* puerarin* treatment. (b) 10 min after *puerarin* treatment. (c) SHRs control group. (d) SHRs receiving *puerarin* for 14 days. (e) SHRs receiving nimodipine for 14 days. A: arteriole; V: venule.

*Puerarin* injection induced significant arteriole dilation in pia mater of SHRs and WKY rats at 3 min after injection, as compared with baseline values (*P* < 0.05) (Table 2). The arteriole relaxation in normotensive WKY rats peaked at 10 min and was sustained to 40 min after *puerarin* injection (*P* < 0.05). In SHRs, the arteriole relaxation peaked at 30 min, when the internal arteriole diameter increased by 26% as compared with baseline (*P* < 0.05). Moreover, SHRs and WKY rats did not differ in extent of arteriole relaxation. The internal diameter of the venule in both groups remained unchanged after* puerarin* treatment (Figures 3(a) and 3(b)).

Laser Doppler perfusion revealed that the baseline level of blood perfusion of pia mater in WKY rats was significantly higher than that in SHRs at baseline (*P* < 0.05) (Table 3). In WKY rats, *puerarin* injection increased blood perfusion in cerebral pia mater by 18% (*P* < 0.05) at 10 min after injection as compared with baseline and decreased to the baseline level at 40 min after injection. In SHRs, blood perfusion was increased by 22% (*P* < 0.05) and 15% (*P* < 0.05) at 10 and 20 min, respectively, after *puerarin* injection as compared with baseline and was reduced to the baseline level at 40 min after *puerarin* injection (*P* > 0.05).

Results from intravital microscopy revealed no significant change in diameter of microvessels after 14-day vehicle treatment in both SHRs and WKY rats as compared with controls, which indicates that propanediol had no effect on microvascular tension in pia mater in SHRs and WKY rats (Table 1). However, *puerarin* treatment for 14 days was associated with a significant increase in internal diameter of arterioles in SHRs. Compared with vehicle-treated and SHRs controls, the arteriole diameter with* puerarin* treatment in SHRs was increased by 29% and 28.8% (*P* < 0.05), respectively (Table 1). *Puerarin*-induced arteriole relaxation was greater than that with nimodipine-treated relaxation. However, laser Doppler perfusion imaging showed that both *puerarin* and nimodipine did not change the blood flow perfusion in cerebral pia mater in all groups (Figures 3(c)–3(e)).

### 3.5. *Puerarin* Treatment Induced MECs Proliferation

**Figure 4:**
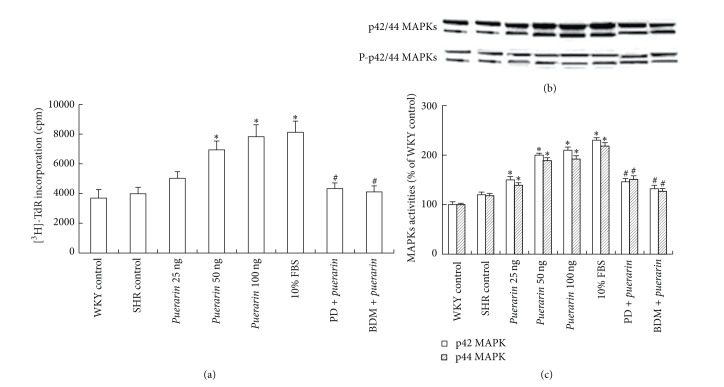
Effect of *puerarin *on proliferation and activity of p42/44 mitogen-activated protein kinases (MAPKs) in microvascular endothelial cells (MECs). (a) MECs proliferation detected by [^3^H]-TdR incorporation. (b) Western blot analysis of phosphorylation of p42/44 MAPKs in MECs (upper panel). Equal protein loading was verified by reblotting with antitotal p42/44 MAPK antibody (lower panel). (c) Densitometry of the immunoblots shown in (a). Data are mean ± SD, *P < 0.05 compared with SHRs control, ^#^ *P* < 0.05 compared with *puerarin* treatment [100 ng], and *n* = 3.

SHRs and WKY control rats did not differ in [^3^H]-TdR incorporation in MECs at 24 h (*P* > 0.05) (Figure 4(a)). However, compared with vehicle treatment, *puerarin* treatment (50 and 100 ng/L) induced a 74.1% and 96.5% increase, respectively, in [^3^H]-TdR incorporation (*P* > 0.05) in SHRs. [^3^H]-TdR incorporation with 10% FBS was increased by 103.9% as compared with SHRs control (*P* < 0.05), which was similar to *puerarin*, 100 ng (*P* > 0.05). Pretreatment with PD98059, an inhibitor of the MAPK kinase MEK-1/2 (upstream activators of p42/44 MAPKs), or BDM (an activator of protein phosphatase) abolished *puerarin*-induced proliferation in MECs.

### 3.6. *Puerarin* Treatment Activated p42/44 MAPKs in MECs

The activity of p42/44 MAPKs was detected by western blot analysis with an antibody against phospho-Tyr-204. Treatment with *puerarin* (25, 50, 100 ng/L) resulted in 25.0%, 66.7%, and 75.0% increase, respectively, in phosphorylation of the p42 MAPK isoform in MECs and 17.8%, 60.2%, and 62.7% increase in phosphorylation of the p44 MAPK isoform, as compared with control treatment (*P* < 0.05) (Figures 4(b) and 4(c)). The activity of p42- and p44-MAPK with 100 ng *puerarin* was similar to that with FBS (*P* > 0.05). PD98059 or BDM abolished the activation of p42/44 MAPKs induced by* puerarin* (Figures 4(b) and 4(c)).

## 4. Discussion

Hypertension induces spasm, stiffening, and remodeling of resistance vessels, which results in autoregulatory dysfunction in brain arterioles [3]. Along with the elevation of systemic blood pressure, damaged vessels undergo hyperperfusion, which leads to hyperemia, edema, and hemorrhage in cerebral tissue. However, decreased blood pressure caused by antihypertensive drugs may cause hypoperfusion and induce cerebral damage and even stroke. It is urgently needed to identify novel therapies for effective antihypertension with microcirculation improvement in patients with imminent stroke. *Puerarin* is one of the effective ingredients of *pueraria lobata*, a traditional Chinese medicine, with effects on reducing blood pressure and plasma rennin activities [14] and downregulating mRNA expressions of angiotensin II type I receptor (AT1) and angiotensin-converting enzyme 2 (ACE2) in myocardium in SHRs [4]. In the present study, *puerarin *reduced the elevated blood pressure and prevented arteriole remodeling, edema, and ischemia in cerebral tissue in SHRs. Our finding suggested that a 14-day treatment with *puerarin *protected cerebral microcirculation along with the reduction of systemic blood pressure in SHRs, which agrees with other findings demonstrating that flavonoids of *pueraria* reduced blood pressure in SHRs and prevented the tendency to stroke in stroke-prone SHRs [8].

*Puerarin* injection has been widely employed in China for the treatment of acute ischemic stroke. Evidence has illustrated that *puerarin* confers neuroprotective action through antiapoptosis in cultured hippocampal neurons [15] and cerebral vasodilation, which reduced cerebral cortical blood flow and enhanced anterior and middle cerebral artery blood flow [16]. Our data showed that *puerarin*-induced cerebral-protective effects in SHRs were associated with improvement of pia mater microcirculation, as assayed by measurement of diameter of microvessels by video microscopy, blood perfusion by laser Doppler imaging, and angiogenesis by immunohistochemical staining. *Puerarin* injection relaxed arterioles and increased blood perfusion significantly in rat pia mater in both SHRs and normotensive WKY rats. *Puerarin* treatment for 14 days reduced the blood pressure, which was accompanied by a significant increase in arteriole diameter in pia mater and a notable alleviation of microvessel remodeling in brain characterized by thinness in arteriole tunica media and increase in brain microvessel density. Thus, *puerarin* improved microcirculation by inducing angiogenesis and improving arteriole tensility and elasticity. *In vitro* experiments with MECs showed an increase in proliferation 24 h after *puerarin* treatment. These findings suggest that *puerarin* may have a cerebral-protective effect along with antihypertension by improving the structure and function of microvessels and hence increasing blood perfusion in cerebral tissue.

The intracellular signaling mechanism that mediates proliferation requires MAPKs, which play important roles in the regulation of cellular responses during cell proliferation and stress. Among the 3 distinct MAPK families, p42/44 MAPKs are well known to be regulated by mitogens [17]. Our *in vitro* experiments with MECs demonstrated upregulation of p42/44 MAPK activity 24 h after *puerarin* treatment, which suggests that activation of the p42/44 MAPK pathway is associated with MECs proliferation and hence angiogenesis induced by *puerarin*.

In summary, *puerarin* possesses effects of antihypertension and stroke prevention by improving microcirculation in SHRs, which results from the increase in cerebral blood perfusion both by arteriole relaxation and p42/44 MAPKs-mediated angiogenesis.

## Figures and Tables

**Table 1:** Effect of *puerarin* on mean arterial blood pressure (MAP), blood perfusion, and microvessel diameter in Wistar-Kyoto (WKY) rats and spontaneously hypertensive rats (SHRs).

Groups	Heart rate	MAP	Blood perfusion	Microvessel diameter (*μ*m)	
	(beat/min)	(mmHg)	(PU)	Arterioles	Venule
WKY					
Control	389 ± 21	83.72 ± 7.82	4.89 ± 1.09	42.64 ± 10.43	44.72 ± 9.51
Vehicle	387 ± 12	90.73 ± 19.67	3.33 ± 0.89	44.78 ± 6.04	43.66 ± 4.51
*Puerarin *	391 ± 28	91.51 ± 13.58	4.16 ± 0.18	47.8 ± 10.91	43.78 ± 4.04
SHRs					
Control	401 ± 26	99.80 ± 16.16*	4.43 ± 0.77	42.82 ± 7.69	33.29 ± 8.24
Vehicle	398 ± 38	105.92 ± 22.61	3.84 ± 0.82	42.84 ± 6.83	46.08 ± 15.67
*Puerarin *	382 ± 21	85.56 ± 15.62^#^	3.83 ± 0.89	55.20 ± 15.12^#^	47.20 ± 15.13
Nimodipine	378 ± 35	113.38 ± 10.10^#^	4.76 ± 0.58	49.94 ± 12.74^#^	41.74 ± 7.09

Data are mean ± SD, *n* = 7. PU: perfusion unit. *P < 0.05 compared with WKY control group; ^#^ *P* < 0.05 compared with SHRs vehicle.

**Table 2:** Effect of *puerarin* on internal diameter of pia mater microvessels in rats.

Groups	Baseline (*μ*m)	After *puerarin* injection (*μ*m)						
		3 min	5 min	10 min	15 min	20 min	30 min	40 min
WKY	41.24 ± 10.97	49.76 ± 9.32	50.11 ± 12.78	52.12 ± 12.41*	52.08 ± 12.10*	52.23 ± 14.09*	52.02 ± 13.72*	51.47 ± 17.25*
SHRs	44.43 ± 7.90	46.13 ± 7.29	51.89 ± 7.15*	51.69 ± 11.30*	54.16 ± 11.41*	55.16 ± 10.86*	55.95 ± 9.892	55.70 ± 12.09

Data are mean ± SD, *n* = 7. *P < 0.05 compared with baseline level.

**Table 3:** Effect of *puerarin* on blood perfusion in rat pia mater.

Groups	Baseline (PU)	After *puerarin* injection (PU)			
		10 min	20 min	30 min	40 min
WKY	5.89 ± 1.10	6.96 ± 1.71^∗#^	6.58 ± 1.69	6.40 ± 1.53	6.12 ± 1.47
SHRs	4.94 ± 0.45*	6.02 ± 1.23^∗#^	5.69 ± 1.05*	5.19 ± 1.05*	4.94 ± 0.57*

Data are mean ± SD, *n* = 7. PU: perfusion unit. *P < 0.05 compared with WKY group; ^#^ *P* < 0.05 compared with SHRs control group.

## Abbreviations

SHRs: Spontaneously hypertensive rats
MECs: Microvascular endothelial cells
WKY: Wistar-Kyoto
PMSF: Phenylmethylsulfonyl fluoride
MAP: Mean arterial pressure
BDM: 2,3-Butanedione monoxide
FBS: Fetal bovine serum
ECGS: Endothelial cell growth supplement
p42/44 MAPKs: p42/44 mitogen-activated protein kinases.
